# Feeding the world: improving photosynthetic efficiency for sustainable crop production

**DOI:** 10.1093/jxb/ery445

**Published:** 2019-02-16

**Authors:** Andrew J Simkin, Patricia E López-Calcagno, Christine A Raines

**Affiliations:** 1 NIAB EMR, New Road, East Malling, Kent, UK; 2 School of Biological Sciences, Wivenhoe Park, University of Essex, Colchester, UK

**Keywords:** Calvin–Benson cycle, sink capacity, synthetic biology, yield potential

## Abstract

A number of recent studies have provided strong support demonstrating that improving the photosynthetic processes through genetic engineering can provide an avenue to improve yield potential. The major focus of this review is on improvement of the Calvin–Benson cycle and electron transport. Consideration is also given to how altering regulatory process may provide an additional route to increase photosynthetic efficiency. Here we summarize some of the recent successes that have been observed through genetic manipulation of photosynthesis, showing that, in both the glasshouse and the field, yield can be increased by >40%. These results provide a clear demonstration of the potential for increasing yield through improvements in photosynthesis. In the final section, we consider the need to stack improvement in photosynthetic traits with traits that target the yield gap in order to provide robust germplasm for different crops across the globe.

## Teaser

This review explores the evidence to date that manipulation of the Calvin–Benson cycle, photorespiration, and electron transport can lead to improvement in biomass and seed yield. Future prospects are also discussed.

## Introduction

Over the past 50 years, agricultural yields of our major crops have risen, in keeping with demand. For the most part, these increases came about due to advances in agronomic approaches and classical breeding that have maximized plant architecture and light capture, resulting in higher yielding varieties. However, the year on year increase in yields of the major crops in many parts of the world have plateaued, and new technological solutions must be explored to develop higher yielding varieties, to maintain the supply of food required to meet the needs of the growing population (Fischer and Edmeades, 2010; Ray *et al.*, 2013; Long *et al.*, 2015; Ort *et al.*, 2015). It has been estimated that by 2050 the global population will increase from its current 7.6 billion to 9.7 billion, requiring between a 70% and a 100% increase in the yield of the major food crops due to increases in living standards, an increase in the requirements for plant-based proteins for animal feed (increased meat consumption), and an increase in the requirements for plant-based fuels (WorldBank, 2008; RSOL, 2009; Tilman *et al.*, 2011; Tilman and Clark, 2015; FAO, 2017). Clearing new land to bring it into use for crop production is not a feasible option as there is little quality land available and therefore this approach would require an increase in the use of nutrient and water inputs in order to deliver the yields needed. This would also have negative impacts on marine, freshwater, and terrestrial ecosystems, leading to damage to unique habitats and a decrease in biodiversity (Vitousek *et al.*, 1997; Dirzo and Raven, 2003; Godfray *et al.*, 2010; GOFS, 2011; Godfray, 2014). Furthermore, approximately one-third of all greenhouse gas emissions can be attributed to crop production and additional land clearance for agriculture (Burney *et al.*, 2010). To mitigate environmental damage caused by extensive agriculture and land clearance, it will be necessary to meet global food demands without increasing the amount of cultivatable land, emphasizing the need to improve crop yields. Moreover, such yield improvements will need to be managed in conjunction with global climate change, where atmospheric [CO_2_] levels are expected to increase from 409 ppm to 550 ppm by 2050 (Solomon, 2007; Le Quere *et al.*, 2009).

Our aim is to provide an overview of the current work to improve photosynthetic efficiency. This review explores the impacts of manipulating the Calvin–Benson (CB) cycle, photorespiration, and electron transport on biomass and seed yield, and also reports on some of the unexpected outcomes where negative effects were observed. In the last section, we explore the future opportunities including combining multigene manipulation of photosynthetic carbon assimilation to improve yield potential with traits that target the yield gap.

## Photosynthesis and crop yield

Photosynthesis is the primary determinant of crop yield, and the efficiency by which a crop captures light and converts it into biomass over the growing season is a key determinant of final yield, be that biomass or grain (Long *et al.*, 2006). The maximum yield attainable from a crop has been termed yield potential and can be defined as the maximum yield attainable when the best adapted crop variety is grown, in optimal conditions with no biotic or abiotic stress (Evans and Fischer, 1999). Determinants of yield potential are light availability, light capture, energy conversion, and plant architecture. For our major crops, rice, wheat, and maize, the only one of these four components contributing to yield that is below the potential maximum is energy conversion, which is determined by photosynthetic efficiency (Long *et al.*, 2006; Zhu *et al.*, 2010). However, the efficiency of this conversion of energy to harvestable biomass, given that as much as 50% of fixed carbon is lost to photorespiration under certain conditions, has yet to be adequately explored.

Supporting evidence that increased yields can be obtained by increasing photosynthetic CO_2_ assimilation comes from CO_2_ enrichment studies, which have consistently shown compelling evidence that yields can be increased through improved CO_2_ uptake (Lilley *et al.*, 2001; Miglietta *et al.*, 2001; Calfapietra *et al.*, 2003; Ainsworth and Long, 2005; Gielen *et al.*, 2005; Leakey *et al.*, 2009; Weigel and Manderscheid, 2012). Although a number of studies showed that there was a negative correlation between leaf area photosynthesis and yield (Evans, 1993, 1998), in the case of wheat, a positive relationship between photosynthetic rates and biomass (Kruger and Volin, 2006) and yield (Fischer *et al.*, 1998) has been observed.

Direct manipulation of the CB cycle in a variety of species between 1992 and 2015 has demonstrated that even small decreases in a limited number of CB cycle enzymes could have a negative impact on carbon assimilation and growth. For example, reductions in enzymes such as sedoheptulose-1,7-bisphosphatase (SBPase; EC 3.1.3.37; Harrison *et al.*, 1998, 2001; Lawson *et al.*, 2006), the chloroplastic fructose-1,6-bisphosphatases (FBPase; EC 3.1.3.11; Koßmann *et al*., 1992; Sahrawy *et al.*, 2004; Rojas-González *et al.*, 2015), or fructose-1,6-bisphosphate aldolase (FBPA; EC 4.1.2.13; Haake *et al.*, 1998, 1999) resulted in slower growth and a decrease in final biomass yield. Furthermore, a decrease in the activity of the plastid transketolase (TK; EC 2.2.1.1) by 20–40% in antisense tobacco plants was also shown to inhibit ribulose-1,5-bisphosphate (RuBP) regeneration and photosynthesis (Henkes *et al.*, 2001); as light levels increase, the inhibition of photosynthesis became more pronounced with the maximum rate of photosynthesis limited under both saturating light and saturating CO_2_. In TK antisense cucumber, a decrease in growth, net photosynthetic rate, stomatal conductance, transpiration rate, and the number of female flowers per plant was observed (Bi *et al.*, 2015). Taken together, these transgenic studies revealed that there is no single limiting step in photosynthetic carbon assimilation, and that control of flow of CO_2_ in the CB cycle is shared between all of the enzymes. Furthermore, this work also demonstrated that this share of control between enzymes is not equal and that the control exercised by any individual enzyme is dependent on environmental conditions and development stage. The hypothesis from this is that improvements in photosynthesis could be achieved through manipulation of more than one individual step in the CB cycle (Stitt and Schulze, 1994; Raines, 2003).

**Fig. 1.**
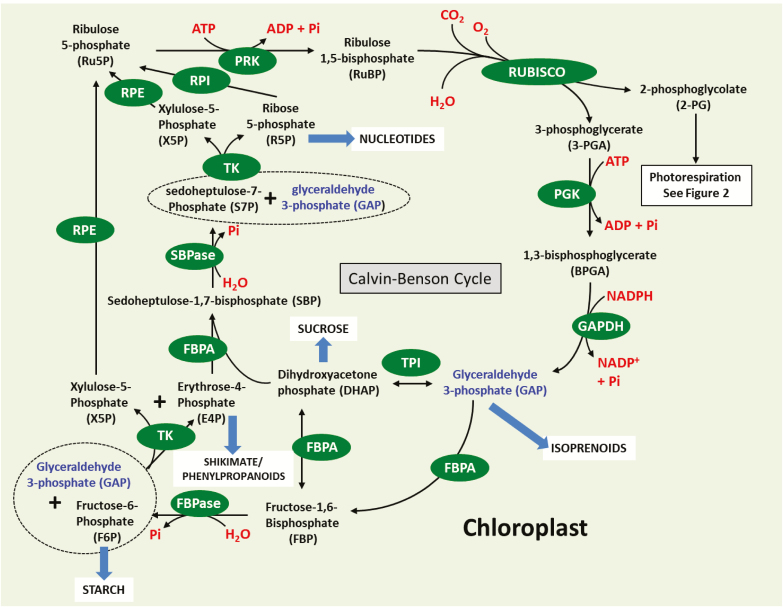
Schematic representation of the Calvin–Benson cycle. Sedoheptulose-1,7-bisphosphatase (SBPase: EC 3.1.3.37), fructose-1,6-bisphosphate aldolase (FBPA: EC 4.1.2.13), fructose-1,6-bisphosphatases (FBPase; EC 3.1.3.11), transketolase (TK; EC 2.2.1.1), phosphoribulokinase (PRK; EC 2.7.1.19), ribulose-phosphate 3-epimerase (RPE; EC 5.1.3.1), triosephosphate isomerase (TPI; EC 5.3.1.1), glyceraldehyde 3-phosphate dehydrogenase (GAPDH; EC 1.2.1.12), phosphoglycerate kinase (PGK; EC 2.7.2.3), ribose 5-phosphate isomerase A (RPI; EC.5.3.1.6), Rubisco (EC 4.1.1.39).

In plants that fix atmospheric CO_2_ using the CB cycle (Fig. 1) enzyme Rubisco, the theoretical maximum energy conversion efficiency attainable is 4.6% for C_3_ plants (Zhu *et al.*, 2010) but, in the field, efficiencies of <50% of this are realized. Modelling studies developed using ordinary differential equations have been used to describe photosynthetic carbon assimilation by the CB cycle and have identified enzymes with the greatest influence on CB cycle CO_2_ assimilation (Laisk *et al.*, 1989; Pettersson and Ryde-Pettersson, 1988; Poolman *et al.*, 2000). The output from the model of Poolman *et al.* (2000) provided evidence that the control of flux in the CB cycle is shared mainly between SBPase and Rubisco, dependent on the environmental conditions in which the plants are grown. Building on these early models, more recent studies have included sucrose/starch biosynthesis and photorespiration leading to the development of a more dynamic model of carbon metabolism (Zhu *et al.*, 2007). The work of Zhu *et al.* (2007) used an evolutionary algorithm together with a model using existing kinetic data and constraining the amount of nitrogen. Based on this, it was proposed that increasing SBPase, FBPA, and ADP-glucose pyrophosphorylase (AGPase; EC 2.7.7.27) in the same plant, together with a modest reduction in photorespiration, could lead to an increase in the efficiency of photosynthetic carbon assimilation. The importance of this model is that it highlighted the fact that more than one target is likely to be needed and that modelling has the potential to allow the most promising combination of targets to be identified. This model remains to be fully tested experimentally.

## Evidence that transgenic manipulation of photosynthesis could increase yield

Early work to improve photosynthetic efficiency through transgenic manipulation focused on the overexpression of a single individual enzyme in the CB cycle (Table 1). The overexpression of SBPase, for example, in *Arabidopsis thaliana* (Arabidopsis) (Simkin *et al.*, 2017*a*), tobacco (Lefebvre *et al.*, 2005; Simkin *et al.*, 2015), and tomato (Ding *et al.*, 2016) has shown that an increase in SBPase enzyme activity results in an increase in both photosynthetic carbon assimilation and biomass yield. Lefebvre *et al.* (2005) showed in tobacco that the photosynthetic CO_2_ assimilation rates increased in young expanding leaves, and both sucrose and starch accumulated, resulting in a 30% increase in biomass. However, no significant increase in photosynthetic rates was observed in the fully expanded leaves of these same plants (Lefebvre *et al.*, 2005). The results observed by Lefebvre *et al*. were confirmed in follow-up experiments showing that these increases were conserved across generations, 10 years apart, and when grown under both high and low light (Simkin *et al.*, 2015). Subsequently, increased SBPase activity in tobacco was shown to increase biomass yield substantially in field-grown tobacco under an open-air elevation of CO_2_ (Rosenthal *et al.*, 2011). Further support for these results also comes from work on Arabidopsis where a 42% increase in biomass, increased CO_2_ assimilation, and accelerated development were observed (Simkin *et al.*, 2017*a*); and in tomato, where biomass, sucrose, and starch all accumulated (Ding *et al.*, 2016), demonstrating that SBPase is one of the enzymes that can exert control over the flow of carbon in the CB cycle in a number of different species. Although the simplest explanation for the positive effect of increasing SBPase activity is a direct consequence of improving CO_2_ assimilation, an interesting alternative hypothesis is that changes in metabolites caused by increasing SBPase activity may act as a signal that alters plant growth and development. The metabolic changes that occur in response to changing SBPase activity have not been elucidated, but it is interesting to note the changes in development which occurred in the SBPase antisense plants and in response to manipulation of other metabolic pathways (Lawson *et al.*, 2006; Raines and Paul, 2006).

**Table 1.** Summary of single targeted manipulations of Calvin–Benson cycle enzymes and their biological outcomes

Manipulation	Transgene expressed	Plant	Functional description	Biomass and yield	References
**Calvin–Benson cycle**	SBPase	Arabidopsis	Tissue-specific expression. 37–85% increase in SBPase, activity, 37% increase in CO_2_ assimilation	42% increase in dry weight and a 53% increase in seed yield	Simkin *et al.* (2017*a*)^*a*^
		Tobacco	Constitutive expression. 90–110% average increase in SBPase activities, increase in photosynthetic rates, increases in sucrose and starch	30–34% increase in dry weight	Lefebvre *et al.* (2005); Simkin *et al.* (2015)^*b*^
		Tomato	Constitutive expression. 55–139% increase in SBPase activity, ~25% increase in CO_2_ assimilation, increases in sucrose and starch	Up to 39% increase in dry weight in best lines. Tomato plants found to be more chilling tolerant	Ding *et al.* (2016) ^*b*^
		Wheat	Constitutive expression. Up to 90% increase in SBPase activities in some lines, increase in CO_2_ assimilation	Up to 40% increase in grain yield	Driever *et al.* (2017) ^*b*^
		Rice	Constitutive expression. Up to 200% increase in SBPase activities in some lines, increased CO_2_ assimilation rates under elevated temperature	Higher growth rates under elevated temperature	Feng *et al.* (2007) ^*b*^
	Cyanobacterial SBPase	Tobacco	Tissue-specific expression. More than 20% increase in the rate of photosynthetic CO_2_ fixation	50% increase in final dry weight	Tamoi *et al.* (2006) ^*a*^
	Cyanobacterial FBPase	Tobacco	Tissue-specific expression. 15% increase in CO_2_ fixation rates in some lines	30% increase in dry weight	Tamoi *et al.* (2006) ^*a*^
	FBPaldolase	Arabidopsis	Tissue specific expression. 46–80% increase in FBPaldolase activity, 31% increase in CO_2_ assimilation	32% increase in dry weight, 35% increase in seed yield	Simkin *et al.* (2017*a*)^*a*^
		Tobacco	Tissue-specific expression. 40–90% increase in FBPaldolase activities, 19% increase in photosynthetic CO_2_ fixation	10–30% increase in dry weight at ambient CO_2_ with a 70–120% increase in high CO_2_	Uematsu *et al.* (2012) ^*a*^
	Transketolase	Tobacco	Constitutive expression. 76–150% increase in transketolase activity, no increase in photosynthesis	Negative effect on plant growth resulting in leaf chlorosis	Khozaei *et al.* (2015) ^*b*^
		Rice	Tissue-specific expression. 80–94% increase in transketolase content, no effect on photosynthesis	No changes to biomass, plant height, or tiller number. Chlorosis NOT observed	Suzuki *et al.* (2017) ^*a*^
	Cyanobacterial SBP/ FBPase	Tobacco	Tissue-specific expression. 70% increase in FBPase acitivity, 130% increase in SBPase acitivity, 20% increase in photosynthetic CO_2_ fixation	Increase in biomass of 40–50%	Miyagawa *et al.* (2001) ^*a*^
		Lettuce	Tissue-specific expression. Photosynthetic capacity was increased by 30–60%	60% increase in fresh weight	Ichikawa *et al.* (2010) ^*a*^
	Cyanobacterial SBP/ FBPase	Soybean	Constitutive expression. 4–14% increase in CO_2_ fixation rates in some lines	Under ambient CO_2_, elevated temperature led to reductions in seed yield. Under elevated CO_2_ and elevated temperature, seed yield was maintained while the WT showed 11% and 22% reductions	Köhler *et al.* (2017) ^*c*^

Transgenes were under the control of either photosynthetic tissue-specific promoters or a constitutive promoter.

Growth conditions are indicated: ^*a*^ controlled environmental conditions; ^*b*^ greenhouse; ^*c*^ field experiments.

More recently it was shown that by increasing SBPase activity in wheat, significant increases in photosynthetic rates can be achieved (Driever *et al.*, 2017). Importantly, these increases in SBPase activity resulted in an increase in grain yield (+30–40%) as well as biomass yield. To confirm these results, Driever *et al.* (2017) grew these plants under two different growth regimes. In one experiment, plants were grown at high density where tillering is limited and, in another, at lower density where tillering was encouraged. Under the higher growing density, plants had fewer tillers with an increase in seed number per ear and, at the lower growing density, plants produced more ears with no significant increase in the number of seeds per ear. In the lines with the highest SBPase activity, total seed weight, seed number, and whole-plant biomass were shown to be increased in both experiments, demonstrating that increasing SBPase activity can have a positive effect regardless of planting density (Driever *et al.*, 2017).

The results obtained by overexpression of plant enzymes are supported by parallel research, in which cyanobacterial enzymes SBPase (cySBPase), FBPase (cyFBPase), or the bifunctional fructose-1,6-bisphosphatases/sedoheptulose-1,7-bisphosphatase (cyFBP/SBPase) were expressed in higher plants. In tobacco, Tamoi *et al.* (2006) demonstrated that plants expressing cySBPase showed an increase of >20% in the rate of photosynthetic CO_2_ fixation and in their growth rate, with a 1.5-fold increase in final biomass. Furthermore, some plants expressing cyFBPase were also shown to accumulate a larger amount of biomass (+1.3-fold) compared with controls, with a 15% increase in CO_2_ fixation rates in some lines. These results suggest that both SBPase and FBPase enzyme activities exert some level of control over the flow of carbon through the CB cycle. Moreover, Miyagawa *et al.* (2001) expressed the cyanobacterial cyFBP/SBPase in tobacco cv. Xanthi. These authors showed that the expression of this bifunctional enzyme (FBPase activity increased by as much as 1.7-fold and SBPase activites by 2.3-fold) in plants enhances photosynthesis by ~1.20-fold, increasing biomass by 40–50% (Table 1). These results are supported first by the expression of cyFBP/SBPase in lettuce which resulted in a 1.3-fold increase in photosynthetic capacity and a 1.6-fold increase in fresh weight (Ichikawa *et al.*, 2010) and, secondly in soybean, which showed that plants had significantly higher rates of carbon assimilation (4–14%) compared with controls (Köhler *et al*., 2017).

In 2012, the overexpression of the CB cycle enzyme FBPA in tobacco also resulted in an increase in photosynthesis and growth, under elevated CO_2_ (700 ppm) (Uematsu *et al.*, 2012). Under these conditions, Uematsu *et al*. saw a 1.4- to 1.9-fold increase in aldolase activities, a 1.5-fold elevation of photosynthetic CO_2_ fixation, and an increase in biomass of 70–120%. However, these effects were much less when plants were grown in ambient CO_2_ where increases in biomass ranged from 10% to 30% compared with the wild type (Uematsu *et al.*, 2012).

These individual gene manipulations have demonstrated that increases in the activity of enzymes of the CB cycle can increase photosynthetic carbon assimilation, enhance growth, and lead to significant increases in vegetative biomass under controlled conditions.

## Photorespiration

**Fig. 2.**
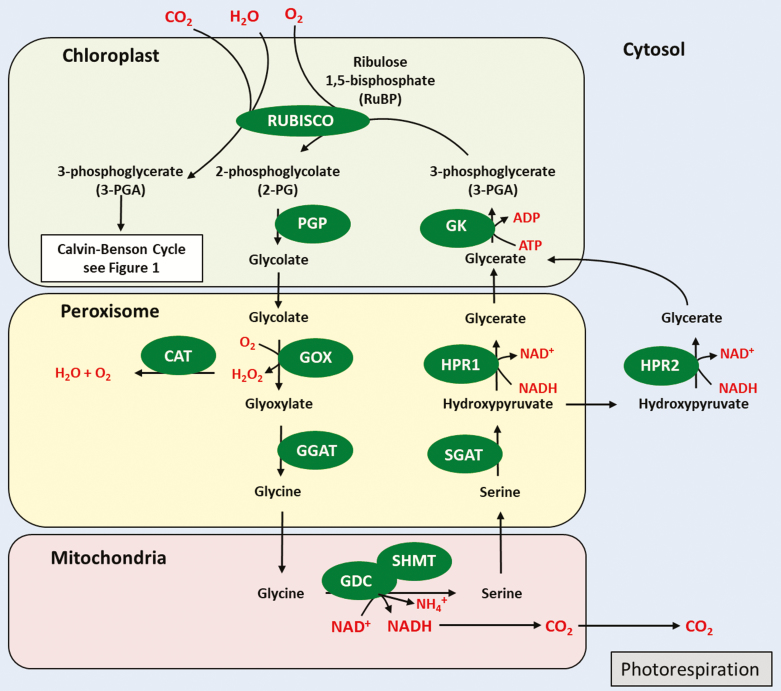
Schematic representation of photorespiration. Glycolate oxidase (GOX; EC 1.1.3.1), 2-phosphoglycerate phosphatase (PGP; EC 3.1.3.13), serine-glyoxylate transaminase (SGAT; EC 2.6.1.45), glycine:2-oxoglutarate aminotransferase (GGAT; EC 2.6.1.4), glycerate-3-kinase (GK; EC 2.7.1.31), hydroxypyruvate reductase (HPR; EC 1.11.81), glycine decarboxylase (GDC), catalase (CAT; EC 1.11.16), serine hydroxymethyltransferase (SHMT; EC 2.1.2.1), Rubisco (EC 4.1.1.39).

In addition to the carboxylation reaction carried out by Rubisco, in which CO_2_ is added to RuBP resulting in flow of carbon through the CB cycle, a competing reaction of the Rubisco enzyme results in the fixation of O_2._ This oxygenase activity of Rubisco competes with the fixation of CO_2_ at the active site (see Fig. 2) and, in ~25% of the reactions, oxygen is added to RuBP instead of CO_2_, leading to the formation of a molecule of 3-phosphoglycerate (3PGA) and a molecule of 2-phosphoglycolate (2PG) at the cost of one ATP and one NAD(P)H. The metabolite 2PG is not used in the CB cycle and needs to be recycled at a high energy cost, thereby reducing the efficiency of CO_2_ assimilation and impacting significantly on yield (Anderson, 1971; Bowes *et al.*, 1971; Ludwig and Canvin, 1971; Sharkey, 1988; Tolbert, 1997; Zhu *et al.*, 2010; Busch, 2013; Walker *et al.*, 2016*a*, *b*).

In the photorespiratory pathway, 2PG is recycled into 3PGA in a process which takes place in three organelles (chloroplast, peroxisome, and mitochondria) and in the cytosol. This pathway is able to recover 75% of the carbon, with the remaining 25% being released as CO_2_ in the mitochondria (Bauwe and Kolukisaoglu, 2003; Peterhansel *et al.*, 2010). Although photorespiration prevents the accumulation of 2PG and the concomitant inhibition of CB by this metabolite, it does so at a high energetic cost. This process also generates one molecule each of hydrogen peroxide (H_2_O_2_) and ammonia (NH_3_) for two oxygenation events for all steps leading to glycine production (Tolbert, 1979). H_2_O_2_ and NH_3_ have been shown to function as signaling molecules with important roles in plant fitness, including disease resistance and nitrogen assimilation (Rachmilevitch *et al.*, 2004; Taler *et al.*, 2004; Rojas *et al.*, 2012); however, both of these molecules can be toxic if they accumulate to high levels (Peterhansel *et al.*, 2010) and the re-assimilation of NH_3_ via glutamine synthetase and glutamine 2-oxoglutarate aminotransferase also adds to the energetic costs (Wallsgrove *et al.*, 1980; Keys, 2006; Masclaux-Daubresse *et al.*, 2010; Moroney *et al.*, 2013; Huma *et al.*, 2018). For these reasons, photorespiration has been a long-standing target in attempts to improve photosynthesis. As reviewed recently, there has been an array of approaches aimed at engineering photorespiration with the goal of increasing crop productivity (South *et al.*, 2018). Here, we will not discuss approaches aiming to introduce a bypass to photorespiration and will only summarize manipulations in the levels of the endogenous proteins involved in this process, some of which have led to an increase in plant productivity. Two successive reactions taking place in the mitochondria have received a particular amount of attention. First, the oxidative decarboxylation of glycine to methylene tetrahydrofolate (THF) by the glycine cleavage system (GCS), which in turn is used by serine hydroxymethyltransferase (SHMT) to form serine from a second glycine (Foyer *et al.*, 2009; Bauwe *et al.*, 2010). First discovered in bacteria by Sagers and Gunsalus (1961), the reversible conversion of glycine to serine is crucial to the majority of organisms so far characterized, including cyanobacteria, green microalgae, and plants (Kisaki and Tolbert, 1970; Kisaki *et al.*, 1971; Eisenhut *et al.*, 2008; Kikuchi *et al.*, 2008; Zelitch *et al.*, 2009; Hackenberg *et al.*, 2011). These reactions involve three different enzymes: the pyridoxalphosphate-dependent enzyme glycine decarboxylase (P-protein; EC 1.4.4.2), the THF-dependent enzyme aminomethyltransferase (T-protein; EC 2.1.2.10), and the NAD^+^-dependent enzyme dihydrolipoyl dehydrogenase (L-protein; EC 1.8.1.4). A fourth protein involved in this process is the lipoic acid-containing H-protein, which acts as a hydrogen carrier and interacts with the P-, T-, and L-proteins, to transfer intermediates successively between the enzymes and finally to NAD^+^ (Oliver *et al.*, 1990; Oliver, 1994; Faure *et al.*, 2000; Douce *et al.*, 2001; Foyer *et al.*, 2009; Bauwe *et al.*, 2010).

Modelling studies indicated that in environments where few stresses are likely, a modest reduction in the levels of the photorespiratory proteins could lead to a better nitrogen distribution, which would in turn lead to higher CO_2_ assimilation (Zhu *et al.*, 2007). However, previous studies had shown that reductions in the flux through the photorespiratory pathway under high photorespiratory conditions (i.e. high temperature or water stress) leads to a decrease in photosynthetic efficiency. For example, knockdown of the GCS P-protein in potato and H-protein in rice was shown to lead to reductions in flux through the photorespiratory cycle, a reduction in the rate at which mitochondria oxidize glycine (–70%), and a reduction in photosynthesis and growth rates (Heineke *et al.*, 2001; Bykova *et al.*, 2005; Zhou *et al.*, 2013; Lin *et al.*, 2016). Antisense P-protein potato plants [containing 30–40% of wild-type (WT) levels] accumulated >100-fold higher levels of glycine and displayed a significant reduction in the rate of glycine oxidation (Heineke *et al.*, 2001; Bykova *et al.*, 2005); and deletion of the GCS P-protein in Arabidopsis was shown to be lethal under non-photorespiratory conditions (Engel *et al.*, 2007). Furthermore, in rice under ambient CO_2_, knockdown of the H-protein also resulted in chlorophyll loss, protein degradation, lipid peroxidation, and an accumulation of reactive oxygen species (ROS), leading to ROS-induced senescence (Zhou *et al.*, 2013). Moreover, a T-protein insertion mutant had very high leaf glycine and glyoxylate levels (Engel *et al.*, 2008) and a T-protein knockout was shown to be lethal even in a non-photorespiratory environment on growth medium; however, a T-protein knockdown (containing ~5% of WT protein levels) was able to grow in normal air, although at a lower growth rate with a lower photosynthetic performance, and accumulated a moderate amount of glycine (Peterhansel *et al.*, 2013*b*). These results are consistent with earlier studies by Wingler *et al.* (1997) who identified a heterozygous barley mutant with a 50% reduction in the levels of the GCS H-protein. When grown in air, no significant difference in metabolites content of photosynthesis was observed in these plants. However, under low CO_2_ and high light where photorespiration is enhanced, photosynthetic rates decreased and glycine accumulated. These plants also showed a 2-fold increase in glycine content and had lower CO_2_ assimilation rates under drought stress (Wingler *et al.*, 1997; Shimada *et al.*, 2008).

Given that a reduction in activity of the enzymes of the photorespiratory pathway resulted in negative effects, the overexpression of components of the GCS has been explored as a strategy to increase photorespiration flow and decrease accumulation of photorespiratory intermediates. In Arabidopsis, overexpression of the H-protein or L-protein resulted in an improvement in photosynthesis and an increased vegetative biomass (Timm *et al.*, 2012, 2015, 2016; Simkin *et al.*, 2017*a*) (Table 2). Furthermore, recent work in tobacco has also shown that the mesophyll-specific overexpression of the H-protein results in enhanced growth and increased biomass both in the greenhouse and when grown in the field (up to 47%) (López-Calcagno *et al.*, 2018). In contrast, overexpression of the T-protein did not alter photosynthetic CO_2_ uptake or improve plant growth in Arabidopsis (Timm *et al.*, 2018). Interestingly, L-protein overexpressors were also shown to have high sucrose (and fructose and maltose) contents (Timm *et al.*, 2015). Timm *et al.* (2015) proposed that the enhanced photorespiratory metabolic capacity of L-protein overexpression alters carbon flow through the tricarboxylic acid (TCA) cycle. Interestingly, the overexpression of one or other of the GCS proteins did not lead to an increase in the other three GCS proteins (Timm *et al.*, 2012, 2015; Peterhansel *et al.*, 2013*a*; López-Calcagno *et al.*, 2018). These results reveal that the biosynthesis of the four GCS proteins is independently regulated and may only be linked by factors such as light or developmental stage. This is consistent with the idea that the GCS proteins do not form a true protein-associated complex (Table 2).

**Table 2.** Summary of single targeted manipulations of photorespiration, electron transport, and putative carbon transport and their biological outcomes

Manipulation	Transgene expressed	Plant	Functional description	Biomass and yield	References
**Photorespiration**	Glycine decarboxylase H-protein	Arabidopsis	Tissue-specific expression. 19% increase in CO_2_ assimilation and elevated photosynthetic electron transport rates compared with controls	50% increase in dry weight, no increase in seed yield	Simkin *et al.* (2017*a*);^*a*^Timm *et al.* (2012)^*a*^
		Tobacco	Tissue-specific expression. Increase in GDC-H protein content. Photosynthetic CO_2_ assimilation rates are increased. Damage to PSII by photorespiratory stress is reduced	13–38% increase in dry weight	López-Calcagno *et al.* (2018) ^*b*,*c*^
		Tobacco	Constitutive expression. Protein accumulated to 3.6- to 7-fold higher in constitutively expressing plants compared with tissue- specific expression	Over 50% reduction in leaf area throughout the early growth phase	López-Calcagno *et al.* (2018) ^*b*^
	Glycine decarboxylase L-protein	Arabidopsis	Tissue specific expression. Have high sucrose fructose and maltose contents. Increased rates of photorespiration and CO_2_ were observed	19–47% increase in dry weight	Timm *et al.* (2015) ^*a*^
	Glycine decarboxylase T-protein	Arabidopsis	No alterations in photosynthetic CO_2_ uptake	No increase in plant growth	Timm *et al.* (2018) ^*a*^
**Electron transport**	Algal Cyt *c*_6_	Arabidopsis	Constitutive expression. 31% increase in CO_2_ assimilation rates	30% increase in plant size	Chida *et al.* (2007) ^*a*^
		Tobacco	Constitutive expression. Higher photosynthetic/ electron transport rates and improved water use efficiency. Significant increases in chlorophyll and carotenoid content	Increased biomass	Yadav *et al.* (2018) ^*a*^
	Rieske FeS	Arabidopsis	Constitutive expression. Up to 30% increase in CO_2_ assimilation, elevated photosynthetic electron transport rates compared with controls. Significant increases in chlorophyll and carotenoid content	29–72% increase in dry weight and up to 51% increase in seeds yield in some lines	Simkin *et al.* (2017*b*)^*a*^
**Carbon transport**	Cyanobacterial inorganic carbon transporter B	Arabidopsis	Constitutive expression. Significantly higher photosynthetic rates	Approximately 23% increase in biomass at low humidity	Lieman-Hurwitz *et al.* (2003, 2005)^*a*^
		Tobacco	Constitutive expression. 20–28% increase in CO_2_ assimilation rates	71% increase in biomass	Simkin *et al.* (2015) ^*b*^
		Rice	Constitutive expression. 18% increase in CO_2_ assimilation	17.9% increase in biomass and increased plant height	Gong *et al.* (2015) ^*c*^
		Soybean	Constitutive expression. Approximately 7–20% increases in photosynthetic CO_2_ uptake	In ambient CO_2_, a 30% increase in dry weight and a 30% increase in seed yield, Up to 35% increase in dry weight and 6% increase in seed mass in elevated CO_2_	Hay *et al.* (2017) ^*b*,*c*^

Transgenes were under the control of either photosynthetic tissue-specific promoters or a constitutive promoter.

Growth conditions are indicated: ^*a*^ controlled environmental conditions; ^*b*^ greenhouse; ^*c*^ field experiments.

It has been demonstrated previously that 2PG is toxic as it has been shown to inhibit two CB cycle enzymes, triose-phosphate isomerase (TPI) in pea (Anderson, 1971), which plays important roles in both the CB cycle and starch synthesis, and PRK in spinach (Kelly and Latzko, 1976). Recent work supporting this hypothesis has demonstrated that 2PG also inhibits the CB cycle enzymes TPI and SBPase, slowing down starch synthesis in Arabidopsis (Flügel *et al*., 2017). Additionally, glyoxylate has been shown to inhibit Rubisco activation in isolated chloroplasts and *in vivo* (Chastain and Ogren, 1989; Campbell and Ogren, 1990; Hausler *et al.*, 1996). These authors have proposed that the additional stimulation of GCS activity results in a reduction in the levels of these photorespiratory metabolites, reducing the possibility of CB cycle inhibition (see Fig. 2) (Anderson, 1971; Kelly and Latzko, 1976; Cook *et al.*, 1985; Chastain and Ogren, 1989; Campbell and Ogren, 1990; Eisenhut *et al.*, 2007; Timm *et al.*, 2012, 2015; Lu *et al.*, 2014; Simkin *et al.*, 2017*a*).

Given the results obtained from both the down-regulation and up-regulation of proteins in the photorespiratory pathway, reducing photorespiration by decreasing the activities of the GCS enzymes has largely been abandoned. To date, the most heavily studied and most promising approaches to enhancing productivity by limiting photorespiration come from the introduction of alternative routes to metabolize 2PG, liberating CO_2_ for use in the CB cycle (Kebeish *et al.*, 2007; Khan, 2007; Carvalho *et al.*, 2011; Maier *et al.*, 2012; Peterhansel *et al.*, 2013*a*, *b*; Nölke *et al*., 2014; Dalal *et al.*, 2015; Xin *et al.*, 2015; South and Ort, 2017; South *et al.*, 2018).

## Electron transport

**Fig. 3.**
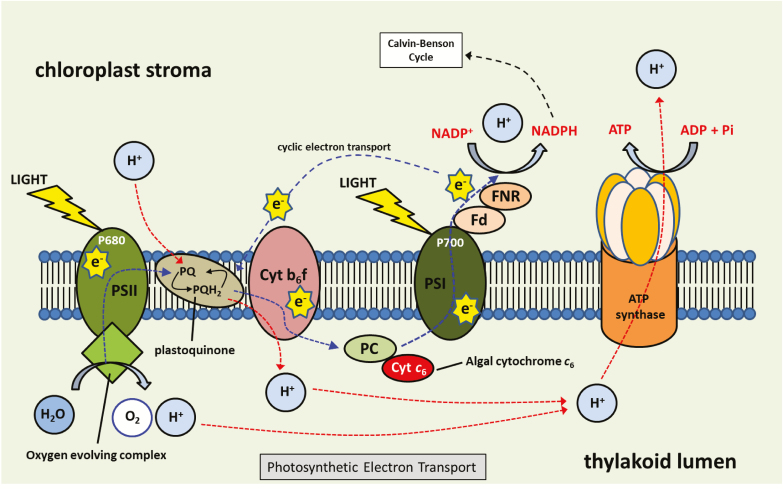
Schematic representation of photosynthetic electron transport. Ferredoxin (Fd), ferredoxin-NADP reductase (FNR), cytochrome *b*_6_*f* complex (Cyt *b*_6_*f*), plastocyanin (PC), cytochrome *c*_6_ (Cyt *c*_6_).

Manipulation of the photosynthetic electron transport chain is another potential option for improving photosynthetic carbon assimilation and yield (see Fig. 3). The first demonstration that increases in electron transport can drive improvements in plant growth came from Chida *et al.* (2007). These authors showed that the expression of the algal (*Porphyra yezoensis*) cytochrome (Cyt) *c*_6_ in the chloroplasts of Arabidopsis leads to an increase in chlorophyll and starch content as well as an increase in ATP and NADPH. These changes were accompanied by an increase in CO_2_ assimilation, efficiency of photosynthetic electron transport, and biomass (Chida *et al.*, 2007). In cyanobacteria and green algae, Cyt *c*_6_ has been shown to replace plastocyanin as an electron transporter in response to copper deficiency (Merchant and Bogorad, 1987). Chida *et al.* (2007) also demonstrated that algal Cyt *c*_6_ can transfer electrons from the Cyt *b*_6_*f* complex to Arabidopsis PSI *in vivo* and at a faster rate than Arabidopsis’s native plastocyanin (Table 2). Similar results were also observed when the Cyt *c*_6_ from *Ulva fasciata* was overexpressed in tobacco (Yadav *et al.*, 2018). These authors observed an increase in photosynthetic rates, improved water use efficiency, and increased growth compared with controls (Table 2).

### The cytochrome *b*_6_*f* complex

The Cyt *b*_6_*f* complex is a central component of photosynthetic electron transport and is located in the thylakoid membrane where it acts in both cyclic and linear electron transport mediating electron flow between PSII and PSI, providing ATP and NADPH for photosynthetic carbon fixation by oxidizing PQH_2_, and reducing plastocyanin (Kurisu *et al.*, 2003; Cramer *et al.*, 2006, 2011; Tikhonov, 2014). The complex is composed of eight different subunits, two encoded in the nucleus [PetC (Rieske FeS) and PetM] and the remaining six [PetA (Cyt *f*), PetB (Cyt *b*_*6*_), PetD, PetG, PetL, and PetN] in the chloroplast genome (Willey and Gray, 1988; Anderson, 1992; Hurry *et al.*, 1996; Knight *et al.*, 2002; Cramer and Zhang, 2006; Cramer *et al.*, 2006; Baniulis *et al.*, 2009; Schöttler *et al*., 2015). This protein complex also functions as a dimer, enhancing its complexity. The transmembrane domains of the cyt *b*_6_ and Rieske FeS proteins are involved in the monomer–monomer interaction of the complex, and the PetD gene product functions as a scaffold, giving these three proteins an important role in the stability of the complex (Hager *et al.*, 1999; Cramer *et al.*, 2006; Schwenkert *et al.*, 2007; Hojka *et al.*, 2014). The PetG, PetN, and PetM subunits have also been shown to have essential roles in both the assembly and stability of the Cyt *b*_*6*_*f* complex, with the *PetL* gene product assigned a minor role in its stability (Bruce and Malkin, 1991; Kuras and Wollman, 1994; Hager *et al.*, 1999; Hojka *et al.*, 2014; Monde *et al.*, 2000; Schöttler *et al.*, 2007; Schwenkert *et al.*, 2007).

Previous studies have shown that by reducing the accumulation of the Rieske FeS protein, it is possible to manipulate the levels of the Cyt *b*_6_*f* complex (Price *et al.*, 1998; Yamori *et al.*, 2011*b*). First, Cyt *b*_6_*f* inhibitors were used (Kirchhoff *et al.*, 2000), and then antisense studies suppressing the Rieske FeS protein (PetC) have shown that the Cyt *b*_6_*f* complex is a key determinant of the electron transport rate (Price *et al.*, 1995, 1998; Hurry *et al.*, 1996; Anderson *et al.*, 1997; Ruuska *et al.*, 2000; Yamori *et al.*, 2011*a*, b). Antisense studies have shown that a decrease in the accumulation of the Rieske FeS protein results in a decrease in photosynthetic electron transport and a reduction in biomass and seed yield (Price *et al.*, 1998; Yamori *et al.*, 2011*b*, 2016). Plants with reduced levels of the Rieske FeS protein were also shown to have a lower Chl *a*/*b* ratio (Hurry *et al.*, 1996; Price *et al.*, 1998), reduced levels of the ATP synthase complex, and a reduction in the transthylakoid pH gradient (Price *et al.*, 1995; Ruuska *et al.*, 2000).

These findings suggested that the electron transport chain, and specifically the Cyt *b*_6_*f* complex, is a limiting step in photosynthetic carbon assimilation and that increasing electron transport could increase photosynthesis and yields. Recognized as a potential target for increasing photosynthetic electron transport, the feasibility of manipulating a membrane-located multiprotein complex has been questioned. In addition to structural complexity of the Cyt *b*_6_*f* complex, the Rieske FeS protein has been shown to be one of the subunits needed for stable assembly of the Cyt *b*_6_*f* complex (Miles, 1982; Metz *et al.*, 1983; Barkan *et al.*, 1986; Anderson *et al.*, 1997), therefore the overexpression of the Rieske FeS protein is a potential route for improving photosynthetic electron flow through the Cyt *b*_6_*f* complex. This was demonstrated by the overexpression of the Rieske FeS protein in Arabidopsis where it was shown to lead to substantial increases in CO_2_ assimilation and relative electron transport rates, and, importantly, to contribute to a 27–72% increase in biomass and up to a 51% increase in seed yield (Simkin *et al.*, 2017*b*) (Table 2). These authors demonstrated using chlorophyll fluorescence imaging and dual-PAM measurements that overexpression of the Rieske FeS protein resulted in an increase in the potential quantum yield of PSII and PSI photochemistry (Genty *et al.*, 1989, 1992; Baker, 2008) from the early stages of development; and that final increases in leaf area evident in mature plants are probably due to a combination of increased photosynthesis and an increase in light capture due to the greater leaf area (Simkin *et al.*, 2017*b*). These data also showed an increase in the fraction of PSII centres available for photochemistry due to observed increases in *q*_L_ and a lower 1–*q*_p_ (Baker *et al.*, 2007). The overexpression of Rieske FeS protein led to an increase in the levels of the two core proteins of the Cyt *b*_6_*f* complex, Cyt *b*_6_ and Cyt *f*, as well as an increase in proteins associated with PSI (LhcaI, PsaA) and PSII (PsbA, PsbD) and the ATP synthase delta subunit (AtpD). Interestingly, a recent study using Arabidopsis reported increases in Cyt *b*_6_*f* complex proteins in plants grown under square wave light compared with plants grown under fluctuating light. These increases in Rieske FeS, Cyt *b*_*6*_, and Cyt *f* proteins were also accompanied by increases in PSI/PSII proteins LhcaI, PsaA, PsbA, PsbD, and AtpD (Vialet-Chabrand *et al.*, 2017). Furthermore, the *hcf* mutant, in which the biogenesis of the Cyt *b*_6_*f* is reduced, was shown to have a decrease in components of both PSI and PSII (Lennartz *et al.*, 2001). HCF164 encodes a thioredoxin (Trx)-like protein, anchored to the luminal side of the thylakoid membrane where it functions as a disulphide oxidoreductase. These studies imply that some as yet unknown mechanism ties changes in electron transport proteins to changes in PSI and PSII proteins. A chloroplast-localized RNA-binding protein (PBR1) has been shown recently to play a role in co-ordinating the biogenesis of the PSI, Cyt *b*_6_*f*, and the NDH complexes (Yang *et al.*, 2016), providing further support for this hypothesis. This co-ordination of regulation requires further study to elucidate the mechanism behind it.

## Multitarget manipulation of photosynthetic carbon assimilation

It has been shown previously that increasing the activity of SBPase and FBPA in transgenic tobacco resulted in an increase in carbon assimilation and biomass yield (Lefebvre *et al.*, 2005; Uematsu *et al.*, 2012; Simkin *et al.*, 2015). More recently, Simkin *et al.* (2015) demonstrated that the simultaneous overexpression of SBPase and FBPA in tobacco resulted in a cumulative increase in biomass (+62% compared with +34% in SBPase alone). This was the first demonstration that multigene manipulation of the C_3_ pathway can lead to greater increases in yield compared with single manipulations. However, in a parallel study in Arabidopsis, no such cumulative impact was observed when SBPase and FBPA were co-expressed (Simkin *et al.*, 2017*a*). These differing results between tobacco and Arabidopsis show that manipulation of the C_3_ pathway may be species dependent, and specific targeted manipulations may need to be identified for different crop plants.

### Simultaneous manipulation of the Calvin–Benson cycle and expression of ictB

It has also been shown that combining the overexpression of CB enzymes (SBPase and FBPA) with the expression of the putative inorganic carbon transporter B (ictB: YP399376) from cyanobacterium *Synechococcus* sp. PCC 7942 (Bonfil *et al.*, 1998; Kaplan and Reinhold, 1999) resulted in a cumulative increase in biomass yield as compared with SBPase, FBPA, or ictB alone. The ictB protein was originally proposed to be involved in HCO_3_^–^ accumulation; however, subsequent work demonstrated that ictB is not a HCO_3_^–^ transporter and its true function remains unknown (Xu *et al.*, 2008; Price *et al.*, 2013). Interestingly, the transformation of ictB into Arabidopsis and tobacco has resulted in significantly faster photosynthetic rates at limiting CO_2_ levels (Kaplan *et al.*, 2001; Lieman-Hurwitz *et al.*, 2003, 2005). Arabidopsis plants expressing ictB from *Anabaena* sp. PCC 7120 showed a similar phenotype (Lieman-Hurwitz *et al.*, 2005), further demonstrating that ictB could significantly alter carbon assimilation rates. Growth experiments further demonstrated that plants expressing ictB grew significantly faster than wild-type plants under low humidity. Lieman-Hurwitz and colleagues proposed that ictB enhances photosynthesis and growth in transgenic Arabidopsis plants due to a higher internal CO_2_ concentration around Rubisco resulting in higher enzyme activity (Lieman-Hurwitz *et al.*, 2003). In rice, the expression of ictB resulted in an 18.4% increase in photosynthetic carbon assimilation and enhanced mesophyll conductance; however, no significant increases in biomass, tiller number, grain number, or grain weight were observed (Gong *et al.*, 2015). The expression of ictB in soybean (*Glycine max* cv. Thorne) was also shown to increase photosynthetic CO_2_ assimilation significantly in both greenhouse and field trials. Plants also showed an increase in biomass production under drought conditions (Hay *et al.*, 2017). Although the function of ictB has not been shown *in planta*, Simkin *et al.* (2015) further demonstrated that the expression of ictB in greenhouse-grown tobacco could result in increases in the maximum rate of CO_2_ assimilation, Rubisco carboxylation (*Vc*_max_), electron transport (*J*_max_), and biomass yield (+71%) compared with controls grown under the same conditions. The analysis of the expression of ictB showed no evidence to support the hypothesis that the stimulation of the carboxylation reaction of Rubisco was the sole the cause of the observed increases in photosynthetic rates given that transgenic plants with increased levels of FBPA and SBPase had similar *A*/*C*_i_ curves to ictB-expressing lines (Simkin *et al.*, 2015).

Although the cumulative effect of the co-expression of ictB with either SBPase or SBPase+FBPA was clear in the biomass data set, no cumulative enhancement of photosynthesis was detected in these plants (Simkin *et al.*, 2015). It should be noted that the analysis of photosynthetic rates in these plants was carried out at a single time point and that the speed of changes during a diurnal period, or at specific times of day, may be greater and cumulative over time in plants expressing multiple transgenes compared with plants expressing ictB alone. This, however, remains to be investigated. The combined expression of ictB with the bifunctional cyFBP/SBPase in rice also resulted in a cumulative increase in photosynthetic rates, tiller number, grain number, or grain weight compared with plants expressing either ictB or cyFBP/SBPase alone (Gong *et al.*, 2015).

### Simultaneous manipulation of the Calvin–Benson cycle and photorespiration

In response to the positive impact of increasing photorespiration in photosynthetic tissue on plant growth in Arabidopsis and tobacco, Simkin *et al.* (2017*a*) explored the possibility that simultaneously increasing photorespiration by overexpression of the GCS H-protein and increasing the activity of two enzymes from the CB cycle (SBPase and FBPA) could have a cumulative impact on photosynthetic efficiency and yield (Simkin *et al.*, 2017*a*). In this work, plants expressing SBPase, FBPA, and GCS H-protein either alone or in combination were evaluated. This study revealed that the simultaneous manipulation of photorespiration and the CB cycle results in a synergistic positive impact on biomass yield under low and high light (Simkin *et al.*, 2017*a*). Interestingly, manipulation of the photorespiratory pathway alone resulted in an increase in biomass yield, but in these plants no increase in seed yield was evident. This is in contrast to results obtained in plants overexpressing CB enzymes where an increase in both biomass and seed yield (+20–39%) was observed. Moreover, simultaneous manipulation of the CB cycle and photorespiratory pathways resulted in a synergistic increase in seed yield (+62%) compared with plants overexpressing CB enzymes alone (Simkin *et al.*, 2017*a*). The reasons for these differential effects on seeds yield are unclear given that plants were grown under the same conditions in a randomized grouping. However, it has been suggested that changes in carbon source/sink allocation lead to changes in starch and sucrose levels observed in GCS H-protein-overexpressing lines (Timm *et al.*, 2012; Simkin *et al.*, 2017*a*). These results further highlight the need to evaluate independent and multitargeted manipulations in different plant species to identify the specific targets to improve crop yields.

## Unexpected outcomes of targeted manipulations

Although this review highlights a number of successes in improving photosynthesis, it should also be noted that not all manipulations have led to beneficial or desired outcomes. Recent work in tobacco carried out by Khozaei *et al.* (2015) showed that constitutive overexpression of the CB cycle enzyme TK led to a negative effect on plant growth and resulted in leaf chlorosis (Table 1). Plants overexpressing both TK and SBPase also demonstrated a mottled phenotype and restricted growth, indicating that overexpressing SBPase in conjunction with TK is not sufficient to overcome the phenotype observed in TK-overexpressing lines (CAR, unpublished data). Furthermore, the overexpression of TK in rice (+80 to 94%), either alone or in combination with the overexpression of Rubisco, did not lead to an increase in photosynthesis or an increase in biomass (Suzuki *et al.*, 2017). The results obtained here have also been observed in cyanobacteria (Liang and Lindblad, 2016). In cyanobacteria, increasing SBPase and FBPase activity has been shown to increase biomass, whilst the overexpression of TK resulted in a chlorotic phenotype, consistent with the observations in tobacco (Liang and Lindblad, 2016). Another example of unexpected outcomes is the constitutive overexpression of the GCS H-protein. In three previous studies, it was demonstrated that the tissue-specific overexpression of the GCS H-protein resulted in an increase in photosynthetic efficiency and in biomass (Timm *et al.*, 2012; Simkin *et al.*, 2017*a*; López-Calcagno *et al.*, 2018). However, López-Calcagno *et al.* (2018) also demonstrated that the constitutive overexpression of the H-protein resulted in a reduction in growth and biomass, with young plants displaying a >50% decrease in leaf area (Table 2). Constitutive overexpression of the GCS H-protein also resulted in a significant decrease in glucose, sucrose, and fructose (59, 24, and 25%, respectively) and a significant increase in starch (39%). Finally, the expression of the bifunctional cyFBP/SBPase in soya led to a significant decrease in seed yield under ambient CO_2_ and elevated temperature compared with the WT (Table 1; Köhler *et al*., 2017). However, in this instance, under elevated CO_2_ and elevated temperature, seed yield was maintained whilst the WT showed an 11–22% decrease, indicating that the manipulation of photosynthesis can result in both positive and negative impacts depending on growth conditions.

## Improving the efficiency of responses to the fluctuating light environment

In nature, plants must be able to respond to fluctuations in light intensity that take place over time periods ranging from seconds to minutes. In the short term, these changes are modulated by a series of regulatory processes, which must allow for a rapid shift from a low to a high photosynthetic rate (Athanasiou *et al.*, 2010; Alter *et al.*, 2012; Kono and Terashima, 2014; Kaiser *et al.*, 2016, 2018a; Yamori, 2016; Vialet-Chabrand *et al.*, 2017; Matthews *et al.*, 2018). It has been shown that under fluctuating light conditions, photosynthesis can be limited during transitions from low to high light and from high to low light. The time taken for photosynthesis to reach steady state following a change in light availability can be between a few minutes and >30 min, dependent on the duration and magnitude of the change regardless of whether there were increases or reductions in light level. Therefore, manipulating electron transport and the CB cycle to enable a rapid response to fluctuations in light availability has the potential to improve crop yield (Lawson *et al.*, 2012; Vialet-Chabrand *et al.*, 2017; Kaiser *et al.*, 2018*b*; Slattery *et al.*, 2018). Two regulatory processes known to impact on responses to fluctuating light are down-regulation of electron transport and light activation of the enzymes of the CB cycle. Below we present some of the current evidence, which highlights the potential of manipulating these processes to increase photosynthetic efficiency.

### The dissipation of excess energy through non-photochemical quenching

Non-photochemical quenching (NPQ), or the dissipation of excess energy in the form of heat, is an important strategy for photoprotection. When the levels of light absorbed by a leaf exceed the leaf’s assimilatory capacity, there is a decrease in the proton conductance of the chloroplast ATPase that rapidly results in a significant decrease in thylakoid lumen pH (Kanazawa and Kramer, 2002; Takizawa *et al.*, 2008). This change in pH activates qE (Horton *et al.*, 1996; Müller *et al*., 2001), which is able to protect the photosynthetic apparatus over short-term fluctuations in light intensity by dissipating the excess absorbed light energy as heat (Grasses *et al.*, 2002; Külheim *et al.*, 2002; Li *et al.*, 2002). The process of NPQ involves the activation of the xanthophyll cycle, which is dependent on the activities of the enzymes violaxanthin de-epoxidase (VDE) and zeaxanthin epoxidase (ZEP) (Demmig-Adams and Adams, 1996), together with sensing of changes in the lumen pH by PsbS, a PSII protein. This process of induction occurs over a time scale of seconds to minutes and is independent of changes in gene expression (Li *et al.*, 2002, 2004). Although changes in NPQ are relatively rapid, they are not instantaneous. This is particularly noticeable in the rate of NPQ relaxation, which can lead to loss of potential photosynthetic capacity, as down-regulation of PSII continues even when light levels have returned to non-stress levels (Pérez-Bueno *et al*., 2008). Recently, Kromdijk *et al.* (2016) modified both components of the NPQ system; increasing the amount of PsbS for pH sensing and the amount of ZEP and VDE for more rapid xanthophyll cycle kinetics. These plants displayed a faster relaxation of NPQ and recovery of CO_2_ fixation rate, and potentially higher photoprotection under excessive light conditions. This manipulation showed that without directly changing photosynthetic capacity, maximum carboxylation capacity (*Vc*_max_), or ribulose bisphosphate regeneration capacity (*J*_max_), the overall CO_2_ fixation of plants exposed to fluctuating light conditions could be improved. Furthermore, plants in these experiments showed a 14–20% increase in biomass under both glasshouse and field conditions (Kromdijk *et al.*, 2016).

### Redox regulation of photosynthesis

The CB cycle is dependent on ATP and NADPH produced by the photosynthetic electron transport chain. It is thus of crucial importance that these two processes, the CB cycle and production of ATP and NADPH, are closely regulated in order to balance CO_2_ fixation with the availability of energy from the light reactions to drive the CB cycle. One of the most important mechanisms to link these processes relies on a group of redox-sensitive molecules, the Trxs. In plants, Trxs were first identified during the 1970s (Wolosiuk and Buchanan, 1977; Buchanan *et al.*, 1979; Wolosiuk *et al.*, 1979; Buchanan, 1980, 1991), and the mechanisms of action of these molecules have been well characterized along with the enzymatic activities they modulate, which includes the CB cycle, the malate valve, and photorespiration (Buchanan and Balmer, 2005; Balsera *et al.*, 2014; Knuesting and Scheibe, 2018; Nikkanen and Rintamaki, 2014; Schürmann, 2003; Schürmann and Buchanan, 2008; Yoshida and Hisabori, 2017). Four types of typical Trxs are reported for chloroplasts, Trx *f*, *m*, *x*, and *y*. Trxs function by transmitting the redox signal from ferredoxin thioredoxin reductase (FTR) to target enzymes. It has been well described that Trx *f* and *m* reductively activate the CB cycle enzymes phosphoribulokinase (PRK), NADP-glyceraldehyde-3-phosphate dehydrogenase (GAPDH), FBPase, and SBPase (Buchanan, 1980; Laing *et al.*, 1981; Wirtz *et al.*, 1982; Crawford *et al.*, 1989; Scheibe, 1991; Geiger and Servaites, 1994; Hutchison *et al.*, 2000; Schürmann and Jacquot, 2000; Howard *et al.*, 2008; Schürmann and Buchanan, 2008; Michelet *et al.*, 2013; Naranjo *et al.*, 2016). The mechanism of dark deactivation of these enzymes on the other hand is not yet well understood, and has not been exploited for improving photosynthesis. Nevertheless, a recently published study suggests that a stroma-localized atypical Trx from Arabidopsis, designated Trx-like2 (TrxL2), could be responsible for oxidatively deactivating the CB enzymes. This might be a novel target to explore if manipulation of this process can impact photosynthetic efficiency (Yoshida *et al.*, 2018).

A chloroplast NADPH-dependent thioredoxin reductase (NTRC) has also described as an important player in stress and oxidative damage responses (Serrato *et al.*, 2004; Pérez-Ruiz *et al*., 2006). Like Trxs, the NTRC can interact with a number of enzymes in the chloroplast including CB cycle enzymes; additionally, it can interact with 2-Cys peroxiredoxins and Trxs, and is activated by both light and NADPH produced in the oxidative pentose phosphate pathway. Given their similarities, it is not surprising that Trx and NTRC have been proposed to have some overlapping functions (Thormählen *et al.*, 2015; Nikkanen *et al.*, 2016).

Although these regulatory mechanisms enable the light activation of the CB cycle, they also impose a limitation as the activation process of Rubisco and the other enzymes of the CB cycle is slower than the change in environmental conditions. Reducing the time it takes to reach maximum steady-state photosynthesis could have a significant impact on the CO_2_ assimilated over the life of every leaf, particularly when considering how dynamic light incidence can be in plant canopies in field settings (Chazdon and Pearcy, 1986*a*, *b*; Krall *et al.*, 1995; Tinocoojanguren and Pearcy, 1995; Lawson *et al.*, 2012; Taylor and Long, 2017).

Limitations imposed by the rate of Rubisco activation are of particular importance in photosynthetic induction (Sage *et al.*, 1987; Hammond *et al.*, 1998; Soleh *et al.*, 2016); a large number of studies have focused on understanding Rubisco and its activation by Rubisco activase (Rca) (Portis and Parry, 2007; Portis *et al.*, 2008; Carmo-Silva *et al.*, 2015). Full activation of Rubisco can take up to 30 min when plants are transferred from the dark into light and, although activation of the remaining CB cycle enzymes via the Trx system occurs more rapidly than for Rubisco, it can still take between 1 min and 10 min. The consequences of these delays in activation of the CB cycle enzymes is that they cause a lag in the time taken for photosynthesis to reach maximum steady-state levels. The impact of this will depend on the magnitude and duration of shade flecks in the natural light environment.

More recently, the consequences of this delay in activation have been shown *in vivo* during sun transitions, where Rubisco activation could limit photosynthesis resulting in a reduction of 20% in carbon assimilation, which over the season could impact substantially on yield (Taylor and Long, 2017). This light activation of Rubisco is in part mediated by the action of activase, and evidence to suggest that this may provide a target to improve this response has come from overexpression studies, which have led to a more rapid Rubisco activation (Table 3) (Fukayama *et al.*, 2012; Yamori *et al.*, 2012). Additionally, enhancing the thermostability of Rca in Arabidopsis has been shown to improve CO_2_ assimilation rates and plant growth under heat stress (Kurek *et al.*, 2007; Kumar *et al.*, 2009). It is possible that optimizing Rca in both amount and regulation has the potential to decrease the limitations in photosynthesis due to Rubisco activation under a fluctuating light environment.

**Table 3.** Summary of manipulations in Calvin–Benson cycle regulatory mechanisms

Manipulation	Gene targeted	Plant	Manipulation detail	Phenotype	References
**Regulatory proteins**	Rca^*d*^	Rice	Tissue-specific expression of the barley Rca	Reduction in Rubisco amountReduction in CO_2_ assimilationIncreased rate of photosynthetic induction by light	Fukayama *et al.* (2012) ^*b*^
		Rice	Tissue-specific expression of the maize Rca	Increased rate of photosynthetic induction by lightIncreased rate of Rubisco activation at high temperature (40 °C)	Yamori *et al.* (2012) ^*a*^
		Arabidopsis	Constitutive expression of a thermostable Rca isoform	Increased rate of Rubisco activationIncreased CO_2_ assimilation, biomass, and seed yield at high temperature	Kurek *et al.* (2007) ^*a*^
		Arabidopsis	Tissue-specific expression of chimeric Rca	Increased rate of Rubisco activationIncreased CO_2_ assimilation, biomass, and seed yield at high temperature	Kumar *et al.* (2009) ^*a*^
	CP12	Tobacco	Antisense down-regulation of CP12 gene family	Reductions in PRK and GAPDH activityReduced photosynthetic CO_2_ assimilationReductions in biomass	Howard *et al.*, 2011*a*, c)^*b*^
		Arabidopsis	KO of *cp12-1* and *cp12-3* plus reductions of expression of *cp12-2* below 20% of WT levels	80% reductions in PRK levelsReduced photosynthetic CO_2_ assimilationOver 50% reductions in biomass	Lopez-Calcagno *et al.* (2017) ^*a*^
		Aublet	Constitutive expression of CP12	Increased biomass, photosynthetic rates, GAPDH, and PRK activitiesIncreased survival, and reduced ion leakage after chilling treatment	Li *et al.* (2018) ^*b*^
		Aublet	Reduced CP12 expression	Reduced biomass, photosynthetic rates, GAPDH, and PRK activitiesReduced survival, and increased ion leakage after chilling treatment	Li *et al.* (2018) ^*b*^
	Trx *f*	Tobacco	Plastidial expression ofTrx *f*	Up to 21% increase in specific leaf weightUp to 5.5-fold increase in fermentable carbohydrates per unit dry weightLower photorespiration rate	Sanz-Barrio *et al.* (2013) ^*a*^; Farran *et al.* (2014)^*c*^; Aranjuelo *et al.* (2015)^*a*^
	NTRC	Arabidopsis	Constitutive expression of NTRC	42–263% increase in dry weightIncreased starchIncreased photosynthesisEnhanced tolerance to photo-oxidative and drought stresses	Toivola *et al.* (2013) ^*a*^; Nikkanen *et al.*(2016)^*a*^; Kim *et al.* (2017)^*a*^
			Null mutant	90% reductions in growth	Toivola *et al.* (2013) ^*a*^

Transgenes were under the control of either photosynthetic tissue-specific promoters or a constitutive promoter.

Growth conditions are indicated: ^*a*^ controlled environmental conditions; ^*b*^ greenhouse; ^*c*^ field experiments.

^*d*^ For an exhaustive list of manipulations of Rca *in vivo*, see Carmo-Silva *et al.* (2015).

A relatively unexplored strategy for increasing photosynthetic carbon assimilation is by directly targeting Trx with the aim of modulating the redox regulation of the Trx-regulated enzymes more rapidly. As reviewed recently by Nikkanen *et al.* (2017), overexpression of Trx *f* or NTRC (Table 3) has been suggested as a viable strategy for increasing productivity, Increased levels of TRX *f* in tobacco have been shown to lead to increases in specific leaf weight, starch, and sugars under both ambient and increased CO_2_ conditions (Sanz-Barrio *et al.*, 2013; Farran *et al.*, 2014; Aranjuelo *et al.*, 2015). Under glasshouse conditions, overexpression of Trx *f* has led to an increase of 1.7-fold in biomass and up to 5.5 times the amount of fermentable carbohydrates, specifically seven times the amount of starch and twice as much sucrose is present in the WT at the end of the growth cycle (Sanz-Barrio *et al.*, 2013). The accumulation of these carbohydrates provides an opportunity to use these plants for production of biofuel. Enzymatic hydrolysis that co-hydrolyse both starch and structural carbohydrates were carried out using both the WT and TRX *f* overexpressors and significant increases in glucose and fructose were found in the Trx plants which, if used for bio-ethanol production, would lead to estimated ethanol yields of almost 10-fold that of WT plants (Sanz-Barrio *et al.*, 2013). Contrastingly, increases in biomass were not reported for field-grown plants, although Trx *f*-overexpressing plants still displayed increased specific leaf weight (>20% compared with the WT), and increases in both starch and soluble sugars of up to 3.6- and 1.7-fold, respectively (Farran *et al.*, 2014). The increase in starch level was also mantained under high CO_2_ conditions (Aranjuelo *et al.*, 2015). Despite these changes, this manipulation did not lead to detectable increases in photosynthetic rates under current CO_2_ levels, and a decrease in *J*_max_ was reported under elevated CO_2_. This may suggest that the changes in carbohydrate accumulation was due to altered allocation rather than an increase in total carbon captured.

Overexpression of NTRC has also been shown to be beneficial for productivity, leading to increases in starch, photosynthesis, and biomass in Arabidopsis. Photosynthetic quantum yield of CO_2_ assimilation under light intensities limiting photosynthesis and light-saturated CO_2_ fixation rate were ~20% higher in NTRC overexpressors. The biomass increases in the NTRC-overexpressing Arabidopsis plants was between 2- and 2.5-fold in plants grown in long and short days, respectively, under 600 μmol m^−2^ s^−1^ light (no significant difference in biomass was observed when plants were grown at 130 μmol m^−2^ s^−1^ light) (Toivola *et al.*, 2013; Nikkanen *et al.*, 2016). Additionally, overexpression of NTRC has been reported to enhance tolerance to oxidative and drought stresses, which are traits of great importance when improving crops for field conditions (Kim *et al.*, 2017).

Another mechanism for redox regulation of the CB cycle is the activation and deactivation of the enzymes PRK and GAPDH through the formation and breakdown of the GAPDH/CP12/PRK complex (Pohlmeyer *et al.*, 1996; Wedel *et al.*, 1997; Scheibe *et al.*, 2002; Graciet *et al.*, 2003, 2004; Boggetto *et al.*, 2007; Carmo-Silva *et al.*, 2011; Howard *et al.*, 2011*b*), which is also dependent on Trxs (Trost *et al.*, 2006; Marri *et al.*, 2009). This complex has been studied extensively *in vitro* (Avilan *et al.*, 2000; Marri *et al.*, 2005, 2009, 2010) and has also been shown to operate *in vivo* as a response to changes in light availability, modulating the deactivation and activation of PRK and GAPDH enzymes more rapidly than by the sole action of Trxs in higher plants (Howard *et al.*, 2008). This mechanism is so effective that studies have proposed that cyanophages have sequestered it and used it to inhibit the CB cycle and redirect carbon flux from this pathway into the pentose phosphate pathway by expressing a CP12 gene in its host (Thompson *et al.*, 2011). Antisense and mutant studies in higher plants have shown that loss of this protein results in lower photosynthetic rates, slow growth, low GAPDH, PRK, and NADP-MDH, activities and reduced levels of PRK protein (Table 3) (Howard *et al*., 2011a, c; López-Calcagno *et al.*, 2017). *In vitro* and *in vivo* studies have proposed CP12 as a potential chaperone for GAPDH and PRK, preventing heat-induced aggregation and deactivation, and providing protecting against oxidative stress and degradation (Erales *et al.*, 2009; Marri *et al.*, 2014; López-Calcagno *et al.*, 2017). Additionally, it was recently reported that CP12 expression might be linked to increased chilling tolerance (Li *et al.*, 2018). This growing body of evidence indicates that the CP12 protein might have other important roles in regulation and maintenance of photosynthesis or even wider metabolism. If regulation of photosynthetic carbon assimilation is to be fully understood, special attention should be paid to this small unstructured protein (Gontero and Maberly, 2012; López-Calcagno *et al*., 2014). Moreover, studies have shown how the enzymes of the CB cycle are also targets of nitrosylation and glutathionylation, two redox post-translational mechanisms (PTMs) whose importance in signalling and regulation has begun to be recognized in the last decade (Meyer *et al.*, 2008; Zaffagnini *et al.*, 2012; Michelet *et al.*, 2013; Rouhier *et al.*, 2015).

Although the regulation of photosynthesis has received attention (Heyneke and Fernie, 2018), it has not been thoroughly exploited for the realization of increased yield potential, and there is still a gap in the knowledge of the fine detail and speed of Trx-mediated redox regulation of photosynthesis. Nevertheless, given the results observed with overexpression of Trx *f* and NTRC, it would be interesting to investigate whether it would be possible to optimize some of these other redox regulatory mechanisms for increased yield under modern agricultural conditions. One aspect to keep in mind though, given the high diversity and heterogeneity described between CP12, PRK, and GAPDH interactions (Howard *et al.*, 2011*b*), is that it will be important to test these strategies on a species-specific basis as evidence would suggest that the regulatory mechanisms to which the CB proteins are subjected, vary in significance between species, and successful strategies in one species might not necessarily work on another.

## Conclusions and further opportunities

Although the potential for improving yield through single and multigene manipulation of different processes in photosynthesis has been clearly demonstrated, it is unlikely that these alone will provide the large increases in yield under all conditions and in all crops species needed to provide for our growing population in the changing global environment. Going forward, what additional approaches will be required to achieve the increase in yield needed to sustain the growing human population? In addition to the targets discussed in this review, it is likely that it will be necessary to stack a number of different traits targeting photosynthesis. This would include, for example, speeding up relaxation of NPQ and reducing photorespiratory losses by introducing new biosynthetic routes to short-circuit this process. The focus of this review has been on photosynthesis, which provides an increase in source capacity, but it is also likely that it will be essential to consider the sink status of the plants where the source capacity has been increased. The source/sink balance has been the subject of two recent papers, and the potential for combining improvements in both source and sink capacities was highlighted (Chang *et al*., 2017; Sonnewald and Fernie, 2018). Improving photosynthesis is an approach which targets increasing yield potential, but it will also be necessary to close the yield gap in order to provide resilience, and this will need improvement in water use efficiency (WUE), nitrogen use efficiency (NUE), and response to biotic and abiotic stresses. Some advances are also happening in these areas. One example is the recently published work showing how changes in the amount of PsbS protein result in changes in the redox state of Q_A_ (Glowacka *et al*., 2018). Changes in the oxidation state of the plastoquinone pool have been proposed to be able to control stomatal movement (Busch, 2014), and the PsbS transgenics experiments showed a linear relationship between stomatal conductance and Q_A_ redox values, decreasing stomatal opening in response to light and increasing WUE. Furthermore, attempts to improve NUE via the overexpression of glutamine synthetase, which resulted in increased biomass and grain yield, in a number of different plants including tobacco, wheat, and rice have been reported (Wallsgrove *et al.*, 1980; Hoshida *et al.*, 2000; Migge *et al.*, 2000; Fuentes *et al.*, 2001; Habash *et al.*, 2001; Oliveira *et al.*, 2002). A number of in-depth reviews of plant nitrogen cost of photosynthesis, nitrogen uptake, and remobilization have been published (Masclaux-Daubresse *et al.*, 2010; Evans and Clarke, 2018).

In order to achieve the ambitious goals required to feed the growing population, new approaches and technologies will be required including new breeding techniques such as genome editing approaches for endogenous genes modification (CRISPR/Cas9; Arora and Narula, 2017; Georges and Ray, 2017; Aglawe *et al.*, 2018; Wilson *et al*., 2019, Preprint) and synthetic biology to produce designer promoters and proteins. The role of modelling in enabling novel targets to be identified will also be crucial given the complexity of the processes involved. To achieve the full potential of these opportunities, the use of new tools, which allow the quick, efficient, and cheap insertion of multiple transgenes into plants, will be paramount (Engler *et al.*, 2008, 2009, 2014; Marillonnet and Werner, 2015; Exposito-Rodriguez *et al.*, 2017), as will be the development of new promoters for use in crop plants, which are currently limited (Mukherjee *et al.*, 2015; Alotaibi *et al.*, 2018). If these opportunities are to be fully exploited, regulations governing the use of genetic modification and genome editing technologies will need to be reviewed.
